# Bacteremia by *Leuconostoc mesenteroides* in an immunocompetent patient with chronic Chagas disease: a case report

**DOI:** 10.1186/s12879-018-3452-7

**Published:** 2018-11-03

**Authors:** Mayra Gonçalves Menegueti, Gilberto Gambero Gaspar, Ana Maria Laus, Anibal Basile-Filho, Fernando Bellissimo-Rodrigues, Maria Auxiliadora-Martins

**Affiliations:** 1 Nursing School of Ribeirão Preto, University of São Paulo, Av. Bandeirantes, 3900 - Monte Alegre, Ribeirão Preto, SP 14049-900 Brazil; 2 Infection Control Comission, Clinics Hospital, Ribeirão Preto Medical School, University of São Paulo, Av. Bandeirantes, s/n - Monte Alegre, Ribeirão Preto, SP 14049-900 Brazil; 3 Department of Surgery and Anatomy, Ribeirão Preto Medical School, University of São Paulo, Av. Bandeirantes, s/n - Monte Alegre, Ribeirão Preto, SP 14049-900 Brazil; 4 Department of Social Medicine, Ribeirão Preto Medical School, University of São Paulo, Av. Bandeirantes, s/n - Monte Alegre, Ribeirão Preto, SP 14049-900 Brazil

**Keywords:** *Leuconostoc mesenteroides*, Bacteremia, Chagas disease, Intensive care setting

## Abstract

**Background:**

The *Leuconostoc mesenteroides* are members of the *Streptococcae* family and currently has been recognized as potential pathogens. This case describes a bacteremia caused by *L. mesenteroides* in an immunocompetent patient affected by Chagas disease.

**Case presentation:**

A 67-year-old female patient with chagasic megaesophagus and megacolon was submitted to a Heller myotomy for achalasia in 2000 and endoscopic dilatation in 2015. Patient was admitted to the Nutrology Ward in May 2016 with protein-calorie malnutrition associated with achalasia and receiving enteral nutrition. In July 2016, the patient underwent a Serra-Doria surgery. In the third postoperative day she presented an important abdominal distension. She was submitted to a new surgical intervention, and then a terminal ileum perforation was detected, leading the surgeon to perform an enterectomy with side-to-side anastomosis. The next day after the surgery (4th postoperative day) the patient presented a decreased level of consciousness (Glasgow coma scale = 8), hypotension and hypoxemia. In two samples of blood cultures there was growth of *Leuconostoc mesenteroides*. Susceptibility pattern was evaluated by the diffusion disk method. The microorganism was susceptible to penicillin, ampicillin, chloramphenicol, erythromycin, and fluoroquinolones, but resistant to rifampin, tetracycline, vancomycin and teicoplanin.

**Conclusion:**

We concluded that infections caused by *L. mesenteroides* is serious and should be considered not only in settings of immunosuppression and prolonged antimicrobial use, but also in immunocompetent patients undergoing surgeries involving the gastrointestinal tract.

## Background

*Leuconostoc mesenteroides* is Gram-positive cocci, catalase negative, facultative anaerobic organism, belonging to the family *Streptococcaceae* [1]. This microorganism is not part of the normal human microbiome, and it is considered potentially pathogenic. However, infections caused by this pathogen are rare and usually affects immunosuppressed patients, such as those submitted to solid organ or bone marrow transplantation, using TNF-inhibitors (tumor necrosis factor), or exposed to central venous catheters, or who had received previous antibiotic therapy, such as vancomycin. Premature birth and low birth weight are also risk factors for *Leuconostoc* sepsis [2–5]. The vast majority of these bacteria are susceptible to erythromycin, minocycline, clindamycin, and carbapenem, but most of them are resistant to beta-lactams^.^ [6] *Leuconostoc* spp. has intrinsic resistance to vancomycin [6]. This microorganism may cause endocarditis [7], urinary tract infections [8], intrabdominal infections [9] and catheter-associated bloodstream infections [10]. There are also case reports of bacterial meningitis and empyema [11] caused by *L. mesenteroides* in patients with severe immunosuppression [12, 13]. Most of the affected patients are children or neonates [3, 4, 6, 9, 12].

In a retrospective study conducted in a tertiary-care hospital six cases of bacteremia due to *Leuconostoc spp.* in children with short bowel syndrome were analyzed, all of them using total parenteral nutrition [6]. Additionally, an outbreak by *L. mesenteroides* that involved 42 patients in the period from July 2003 to October 2004 in a tertiary-care hospital Juan Canejo, Spain parenteral nutrition was detected as the major risk factor [14].

*L. mesenteroides* are not easily identified in clinical microbiology laboratories. Sometimes being reported incorrectly as Enterococcus species or Streptococcus species. The distinction among these different bacteria may exert an important impact in selecting the appropriate antibiotic regimen for the patient’s treatment, because all clinical isolates of *Leuconostoc* have high-level resistance to vancomycin [6].

This case describes a bacteremia caused by *L. mesenteroides* in an immunocompetent patient affected by Chagas disease.

## Case report

A 67-year-old female patient with chagasic megaesophagus and megacolon, without myocardiopathy was submitted to a Heller myotomy for achalasia in 2000 and endoscopic dilatation in 2015. Patient was admitted to the Nutrology Ward in May 2016 with protein-calorie malnutrition associated with achalasia and using enteral nutrition. In July 2016, the patient underwent a Serra-Doria surgery. In the third postoperative day she presented an important abdominal distension. She was submitted to a new surgical intervention, and then a terminal ileum perforation was detected, leading the surgeon to perform an enterectomy with side-to-side anastomosis.

The next day after the surgery (4th postoperative day) the patient presented a decreased level of consciousness (Glasgow coma scale = 8), hypotension and hypoxemia. She was submitted to orotracheal intubation and transferred to intensive care unit (ICU). On ICU admission the patient was sedated and hydrated. The body temperature was 38.5 °C, arterial blood pressure = 104 × 55 mmHg, the heart rate was 101 beats per minute and respiratory rate = 14 breaths per minute. The *cardiac* a*uscultation was normal*, and respiratory auscultation was compromised with adventitious sounds such as rales and crackles especially in the left pulmonary base. A distended abdomen was observed.

Laboratory findings revealed hemoglobin at 8.9 g/dL and hematocrit of 28%. The white blood cells were increased (20,300/mm^3^), with the left shift until myelocytes (7%) with presence of anisocytosis and neutrophils with abundant toxic granulation. The platelets count was 203,000/mm3, urea = 64 mg/dL, creatinine = 0.96 mg/dL, sodium = 160 mmol/L and potassium = 4.2 mmol/L. The C reactive protein was 19.39 mg/dL. At admission, the Acute Physiology and Chronic Health Evaluation II (APACHE II) for the patient was 33 (death risk of 75%). Blood samples were collected and empiric antibiotic treatment was initiated with cefepime and metronidazole focused for intra-abdominal infection. The patient showed a gradual worsening level of consciousness and septic shock with refractory hemodynamic instability unresponsive to fluid or drugs resuscitation. The patient died three days after ICU admission.

## Diagnosis

In two samples of blood cultures, there was growth only of *L. mesenteroides.* The identification of *L. mesenteroides* was done by the VITEK® 2 system, an equipment capable of automatically carrying out the necessary steps for the identification of microorganisms and determination of antimicrobial susceptibility with a primary inoculum prepared from a culture in solid medium standardized by the manufacturer itself the equipment. This system performs a kinetic analysis of the inoculated culture with readings from each test at 15-min intervals. The optical system of the equipment combines a multi-channel fluorimeter and a reading photometer to record turbidity, fluorescence and colorimetric signals. Susceptibility pattern was evaluated by the diffusion disk method. The microorganism was susceptible to penicillin, ampicillin, chloramphenicol, erythromycin, and fluoroquinolones, but resistant to rifampin, tetracycline, vancomycin and teicoplanin.

## Discussion

The unusual feature of this case report is that our patient did not presented any severe immunosuppressive condition, which could enhance the risk of bloodstream infection due to *L. mesenteroides*, as described in the literature by other case reports.

Some authors have suggested the skin to be a possible source for this pathogen, since most affected patients have been exposed to central-venous catheters by the time of developing the bacteremia episode [6]. Other authors mention the possibility of translocation from the gastrointestinal tract, related to the loss of integrity of the mucous membrane [10]. In an outbreak reported among pediatric patients with short bowel syndrome, it was demonstrated that intestinal mucosa changes may be a risk factor for bacteremia due to *Leuconostoc* [6]. So far, there is no consensus about the main source of this pathogen.

Although catheter-related bloodstream infections are the main clinical presentation of *L. mesenteroides* infections, there have been some case reports of meningitis, abscesses, urinary tract infections, peritonitis, and intra-abdominal infections.

Among the predisposing clinical conditions implicated in these cases are chronic renal failure, rheumatoid arthritis, and alcoholic chronic hepatitis. Similarly, the prolonged use of antibiotics, such as cotrimoxazole, β-lactams, aminoglycosides and vancomycin, also has been associated with the occurrence of these infections [6, 9, 14–16].

Thus, in general, *L. mesenteroides* affects immunosuppressed patients, and/or those previously exposed to antimicrobials or central-venous catheter, and none of these contributing factors was observed in this case.

In the present case, loss of integrity of the intestinal mucosa was present as a direct consequence of the chagasic megaesophagus, short bowel syndrome and exposure to intra-abdominal surgery. So, bacterial translocation from the gastro-intestinal tract could be the source of this pathogen.

Despite its low virulence, this microorganism may be considered as a potential pathogen not only among immunocompromised patients suffering acute febrile illness but also in immunocompetent patients undergoing surgical involving gastrointestinal tract. We emphasize that its identification requires special techniques, to be performed in an appropriate microbiology laboratory.

## Conclusion

We concluded that infections caused by *L. mesenteroides* is serious and should be considered not only in settings of immunosuppression and prolonged antimicrobial use, but also in immunocompetent patients undergoing surgeries involving the gastrointestinal tract.

## Abbreviations

APACHE II: Acute Physiology and Chronic Health Evaluation II
ICU: Intensive Care Unit
